# Controlled positioning of analytes and cells on a plasmonic platform for glycan sensing using surface enhanced Raman spectroscopy†
†Electronic supplementary information (ESI) available: Full experimental procedures, nanoscale topographical images of the device, controlled micro-defined functionalization of the platform, schematic illustration of the Raman reporter–glycan interaction, SERS mapping with nanoscale surface morphology of single isolated HeLa cell, statistical SERS error analysis, and SERS vibrational frequencies of 4-MPBA and cell. See DOI: 10.1039/c5sc03332b



**DOI:** 10.1039/c5sc03332b

**Published:** 2015-10-16

**Authors:** Mohammadali Tabatabaei, Gregory Q. Wallace, Fabiana A. Caetano, Elizabeth R. Gillies, Stephen S. G. Ferguson, François Lagugné-Labarthet

**Affiliations:** a Department of Chemistry and Center for Advanced Materials and Biomaterials , University of Western Ontario , London , ON , Canada N6A 5B7 . Email: flagugne@uwo.ca ; Fax: +1 519 661 3022 ; Tel: +1 519 661 2111 ext. 81006; b J. Allyn Taylor Centre for Cell Biology , Robarts Research Institute , Department of Physiology and Pharmacology , University of Western Ontario , 100 Perth Drive St. , London , ON , Canada N6A 5K8; c Department of Chemical and Biochemical Engineering , The University of Western Ontario , 1151 Richmond Street , London , Ontario , Canada N6A 5B9

## Abstract

The rise of molecular plasmonics and its application to ultrasensitive spectroscopic measurements has been enabled by the rational design and fabrication of a variety of metallic nanostructures. Advanced nano and microfabrication methods are key to the development of such structures, allowing one to tailor optical fields at the sub-wavelength scale, thereby optimizing excitation conditions for ultrasensitive detection. In this work, the control of both analyte and cell positioning on a plasmonic platform is enabled using nanofabrication methods involving patterning of fluorocarbon (FC) polymer (C_4_F_8_) thin films on a plasmonic platform fabricated by nanosphere lithography (NSL). This provides the possibility to probe biomolecules of interest in the vicinity of cells using plasmon-mediated surface enhanced spectroscopies. In this context, we demonstrate the surface enhanced biosensing of glycan expression in different cell lines by surface enhanced Raman spectroscopy (SERS) on these plasmonic platforms functionalized with 4-mercaptophenylboronic acid (4-MPBA) as the Raman reporter. These cell lines include human embryonic kidney (HEK 293), C2C12 mouse myoblasts, and HeLa (Henrietta Lacks) cervical cancer cells. A distinct glycan expression is observed for cancer cells compared to other cell lines by confocal SERS mapping. This suggests the potential application of these versatile SERS platforms for differentiating cancerous from non-cancerous cells.

## Abstract 2

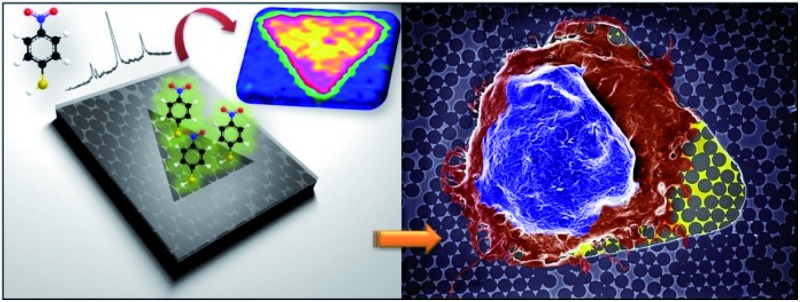
Controlled analyte and cell positioning is enabled on a plasmonic platform with patterned fluorocarbon polymer thin films for SERS-based glycan sensing.

## Introduction

Discovered almost four decades ago, surface enhanced Raman spectroscopy (SERS)^1^ and subsequent techniques such as surface enhanced fluorescence (SEF)^2^ and surface enhanced infrared spectroscopy (SEIRS)^3–5^ have developed into mature methods to give unprecedented levels of sensitivity. SERS in particular provides ultra-high sensitivity down to attomolar concentrations and even to a single molecule level.^3,6–11^ More importantly, the use of these surface enhanced techniques has enabled biosensing and biomolecular recognition with ultra-high sensitivity, opening possibilities for a wealth of applications to probe intimate biological processes with minimal intrusion, better specificity and high reproducibility.^10,12–16^ The interactions between biomolecules and their changes in conformation in response to stimuli are processes that can be probed at the monolayer level with lower light irradiance and shorter acquisition time, thereby reducing experimental invasion and physiological stress.

Keys to the success of surface-enhanced spectroscopies are advances in micro and nanofabrication techniques such as electron-beam lithography and focused ion beam milling that allow one to reproducibly fabricate plasmonic platforms with a 10 nm resolution.^17–22^ The opto-geometric parameters of these platforms can be finely tailored to tune the localized surface plasmon resonance to a selected probe wavelength. Nanosphere lithography is an inexpensive and high throughput technique ideally suited to produce large surfaces of 2D and 3D periodic nanostructures with a variety of shapes such as nanoscale triangles, pyramids, rings, overlaps, gaps, rod chains, and holes.^2,23–26^

Such homogeneous platforms can be further functionalized enabling the study of monolayers of molecules or biomolecules. For example, SERS platforms functionalized with aptamers have been successfully used for toxin and protein recognition.^27,28^ Antibody functionalization of SERS substrates to detect biomarkers of endocrine disrupting compounds was also described.^29^ Furthermore, simultaneous detection and quantification of bacterial pathogens and enzymatic processes such as histone demethylase activity have been probed using SERS-based assays.^30,31^ However, using such platforms, significant challenges are still encountered in the study of biological processes, such as intracellular sensing,^32^ chemical exchanges between cells or responses of cells to endogenous or exogenous stimuli.^33,34^ One significant challenge arises from the inherently random growth of cells over most surfaces.^35^ The positional control of cell growth over an array of plasmonic platforms would open new possibilities for multiplexed parallel screening using SERS, SEF or other optical techniques involving a plasmon resonance that has been tuned to enhance a specific spectral region. Each cell position over a plasmonic platform would be defined by a set of spatial coordinates, allowing automated measurements over a large number of individual cells. This enables acquisition of statistically relevant ensembles of data. The control of cell density over the surface would provide the possibility to control and study cell-substrate and cell–cell interactions.^36,37^ Our group has previously introduced a new method for cell positioning using plasma deposition of fluoropolymer thin films.^35^ However, a plasmonic platform was not incorporated, so it was not possible to perform optical studies of analytes or cells mediated by surface-enhanced methods.

Herein, we introduce the development of a new device that embeds an NSL plasmonic platform into a micro-scale pattern that directs cell adhesion and growth. The micropatterning allows one to locate the analyte on the plasmonic platform and to further perform surface-enhanced measurements with improved sensitivity. We demonstrate that different cell lines such as immortalized cells and neurons can accurately be positioned on such modified surfaces. The functionalization of these platforms with a Raman reporter can also be achieved enabling the detection of other guest molecules. Through functionalization with 4-mercaptophenylboronic acid (4-MPBA), the application of this surface-based device in biosensing is demonstrated by mapping the glycan expression in cell lines including HEK293 human embryonic kidney, C2C12 mouse myoblasts, and HeLa cervical cancer cells.

## Results and discussion

### Preparation and characterization of FC-patterned plasmonic substrates

**Fig. 1:**
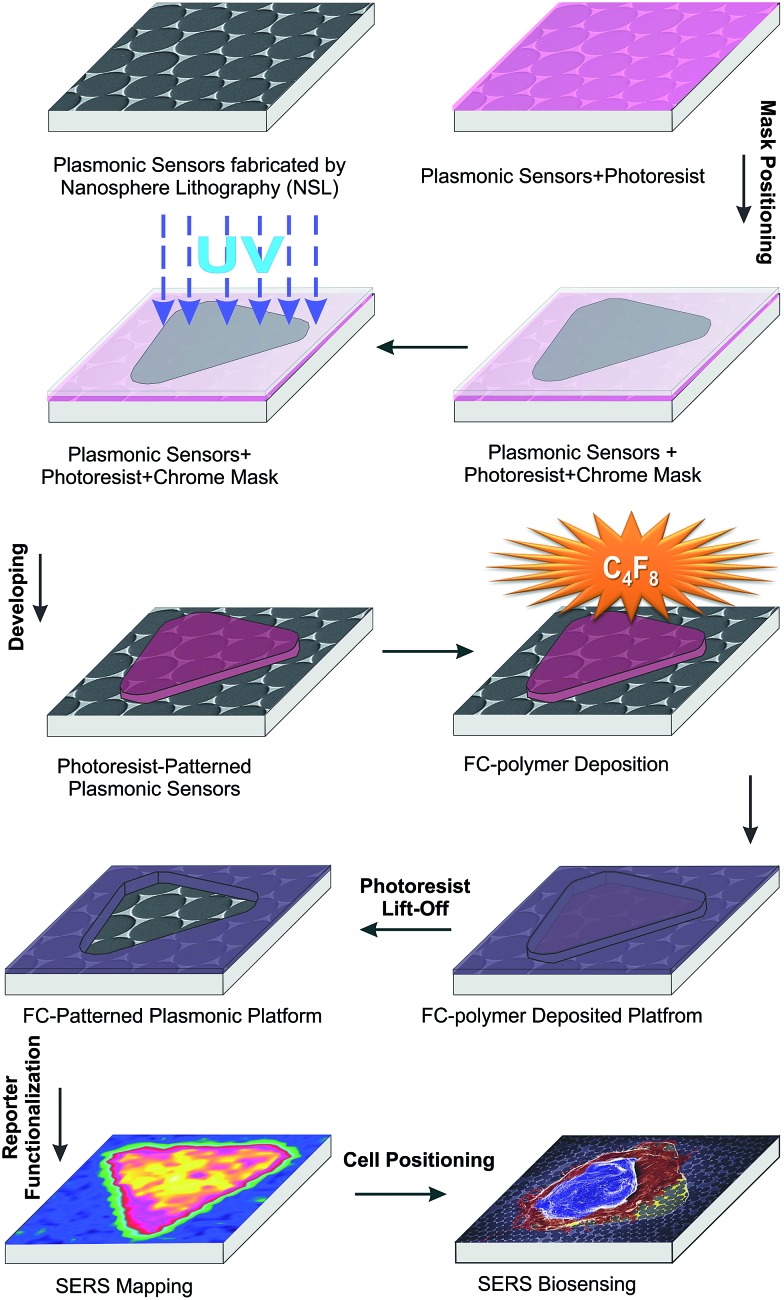
Schematic illustration of the design and fabrication process for the FC-patterned plasmonic platform.

**Fig. 2:**
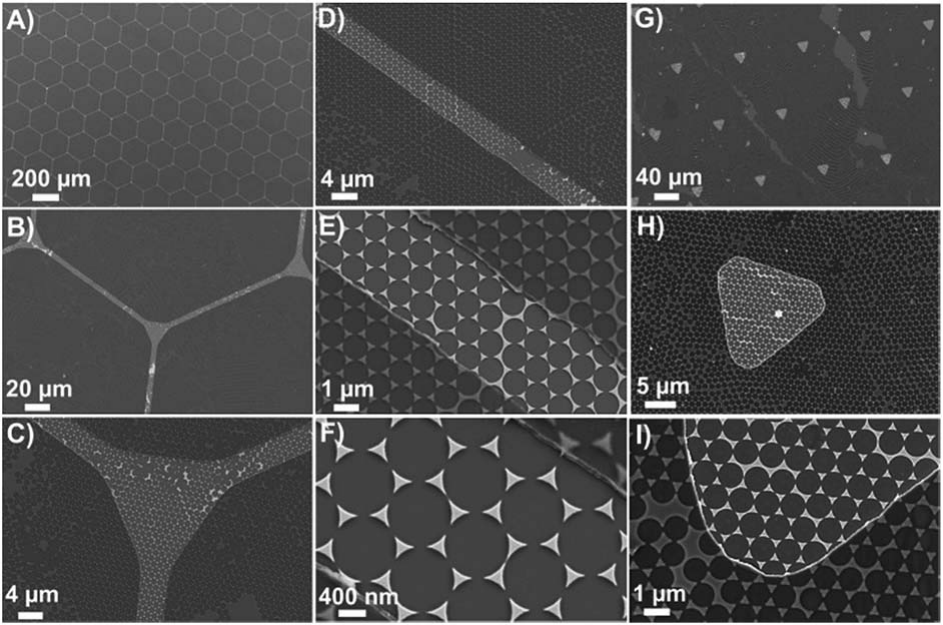
SEM images of FC-patterned plasmonic platform with two different feature patterns: Hexagonal-grid like pattern (A–F) triangular pattern (G–I).

The schematic illustration of our design and fabrication approach for the platform used in this study is depicted in Fig. 1. First, NSL was used to fabricate the SERS platform as previously reported.^38,39^ Next, a photolithographic method with plasma induced fluorocarbon (FC)-polymer thin film deposition was used to provide windows over specific areas on the plasmonic platform for cell growth. Both hexagonal grid-like (Fig. 2A–F) and triangular arrays (Fig. 2G–I) were prepared. The FC-patterned plasmonic platform provides multiple organized sensing nodes that can be tailored, depending on the application. For example, triangular patterns can provide single isolated cells for further biological sensing applications as shown in this work and the organized hexagonal grid like channels can be utilized for interconnected cells such as neurons.

Fig. 2 shows that the sensing windows are surrounded by the thin FC polymer film. The thickness of the FC film was measured by atomic force microscopy (AFM) to be 60 ± 5 nm (Fig. S1, ESI†). The typical sizes of the features are limited to few microns in photolithography. Here we demonstrate in the hexagonal grids that it is possible to fabricate features as small as 4 μm as shown by the width of the channels in Fig. 2C–E. In the case of the triangular patterns, Fig. 2G–I shows that the plasmonic platforms are homogeneously distributed within the FC-polymer patterned regions. Here, the Au nanotriangles serve two purposes. First, the Au surface of the structures can be used for functionalization with a Raman reporter for a target molecule. To demonstrate this, 4-nitrothiophenol (4-NTP) was initially used as a model reporter (Fig. S2, ESI†). Second, the bow-tie assemblies of nanotriangles serve as nanoscale antennas confining electromagnetic (EM) fields in the hot spot regions formed by the facing nanotriangles (Fig. S2, ESI†). Controlled micro-defined functionalization with 4-NTP as a model analyte on this platform is shown in Fig. S2, ESI.† These plasmonic nanotriangles have been shown to have a distribution of gap sizes between 10–200 nm with an average gap of ∼100 nm.^2,25,40^ Such localized enhancement of the EM field is critical to further enhance the Raman signal, providing monolayer sensitivity as well as surface detection of cells placed on nanotriangular plasmonic platform.

### Biocompatibility of FC-patterned plasmonic platform

The biocompatibility of the FC polymer has been demonstrated in previous work, where it was shown that FC-polymer patterning can be efficiently used for controlled cell isolation and proliferation.^35^

**Fig. 3:**
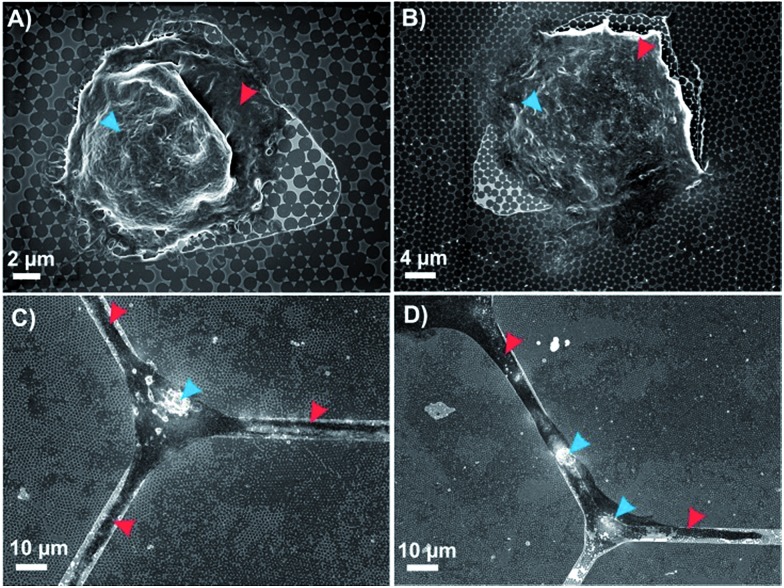
SEM images of positionally-controlled cells on FC-plasmonic platform. (A and B) HEK 293 cells; (C and D) cortical neurons; blue arrows indicate cell nuclei; red arrows indicate cell membranes (A and B) and projections of neurons (C and D).

As the presence of a nanostructure can affect the cell behaviour,^41,42^ in order to ensure that these desirable properties were retained on the FC-patterned plasmonic platform, the culture of human embryonic kidney (HEK 293) cells and mice cortical neurons (14 DIV) on the patterned plasmonic platforms was investigated. As shown in Fig. 3A and B, HEK 293 cells were efficiently isolated in the triangular windows on the plasmonic platform. It was also observed that these cells easily proliferated on the plasmonic nanotriangles.

Similar results were also obtained for the cortical neurons that were grown for 14 days on the hexagonal grid-like patterns. As shown in Fig. 3C and D, the neuronal cell body and its projections were well adapted within the channels on the plasmonic platform. This demonstrates the capability of these substrates to enable controlled cell positioning on the plasmonic platform for further surface enhanced spectroscopic measurements of biomolecules of interest on cells surfaces. Moreover, one can tune the feature patterns and interconnection dimensions with regards to the size of the specific cell line to optimize the sensing conditions.

### SERS activity of FC-patterned plasmonic platform

**Fig. 4:**
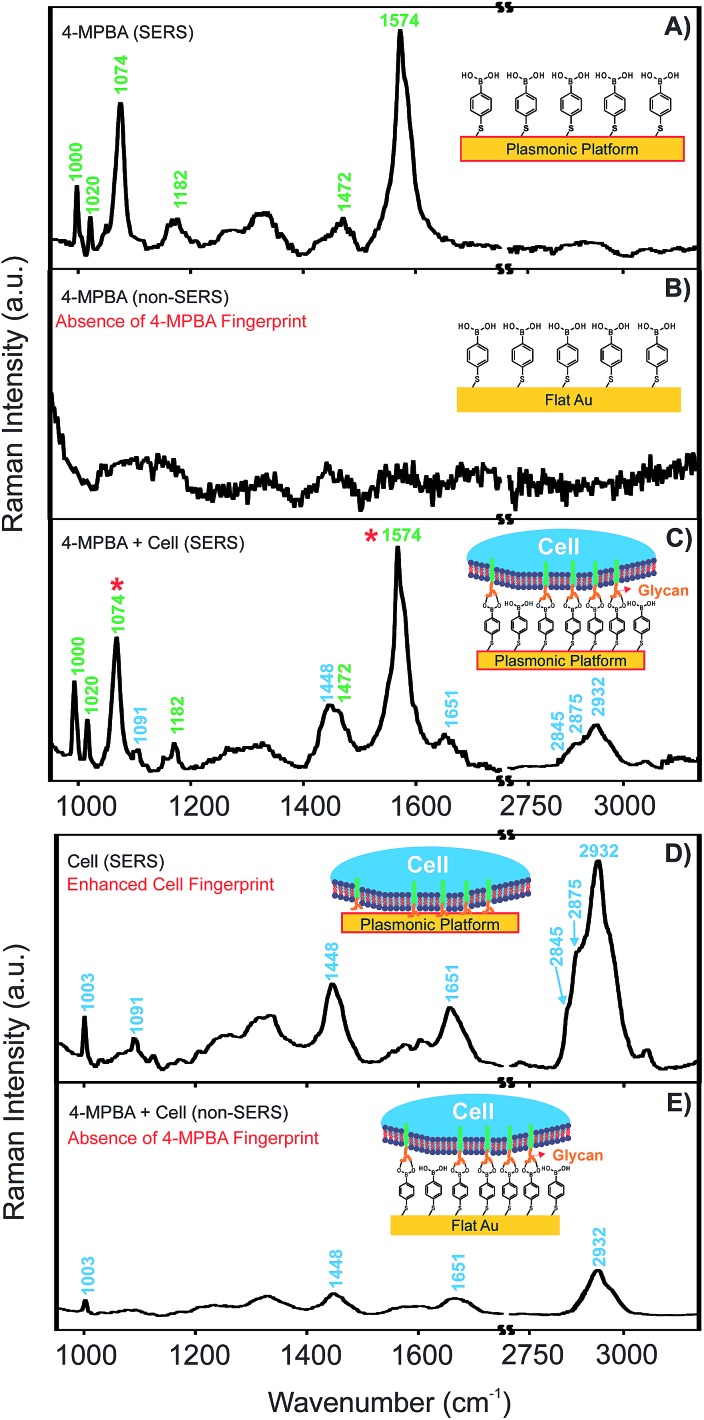
SERS activity of 4-MPBA functionalized FC-patterned plasmonic platform representing 4-MPBA and HEK cell vibrational frequencies (A, C, and D); the non-SERS spectra are obtained on flat Au surface (B, and E) as a control compared to SERS signal obtained on SERS-active plasmonic regions containing nanotriangles within the fluoropolymer. A, B, C are normalized to the same scale. The same process is used for D, and E. Baseline correction is applied to all the spectra.

With the aim of detecting glycans as described below, the Raman reporter molecule was changed to 4-MPBA. Boronic acids have the ability to form cyclic boronate esters with *cis*-1,2 and 1,3 diols, making them prime candidates for binding to and detecting saccharides^43–45^ (Fig. S3, ESI†). 4-MPBA has both a thiol for bonding to the plasmonic nanotriangles and a boronic acid moiety for interaction with glycans on the cell surface. To investigate the SERS activity of the plasmonic platform with respect to this reporter molecule and with respect to cells, SERS spectra of 4-MPBA and HEK 293 cells were collected on FC-patterned plasmonic platforms and on a flat Au surface as a reference and are shown in Fig. 4.

The SERS activity of the platform for 4-MPBA is shown in Fig. 4A, where characteristic peaks corresponding to the vibrational fingerprint of 4-MPBA are clearly observed. As shown in Fig. 4B, the spectrum of 4-MPBA bound to the flat Au used as the control surface lacks the spectral features of the reporter. By introducing the cell on the platform, spectral features of both the Raman reporter and cell were detected as shown in Fig. 4C. Clear spectral features of cellular components appeared on the SERS spectrum obtained on the plasmonic platform without 4-MPBA as shown in Fig. 4D.

When the cell was located on a flat Au surface functionalized with the Raman reporter, the fingerprint of the reporter was absent (Fig. 4E), confirming that a flat Au surface cannot be used for sensing and that the plasmonic platform is indeed required. However, a weak Raman fingerprint of the cell was detected. This cellular fingerprint is due to the thickness (micrometers) of the cell on the Au surface and thus the relatively large amount of cellular material available for detection; the signal is almost certainly not generated from the surface as in the case of a monolayer of the Raman reporter, it is not possible to obtain the signal.

The peaks obtained in spectra of the cells at 1003, 1091, and 1651 cm^–1^, and the group of 2845, 2875, 2932 cm^–1^ can be assigned to the phenylalanine of proteins and lipids, symmetric dioxy stretch of the phosphate backbone, amide 1 C=O and C=C stretching, and CH_2_ stretching of protein and lipid functional groups (Table S1, ESI†).^46^ In the case of the SERS spectrum of 4-MPBA, the changes in two main peaks (labelled with red stars in Fig. 4C) upon interaction with cell surface glycans are assigned to B–OH stretching (1074 cm^–1^) and C–C stretching of the phenyl group (1574 cm^–1^). These two peaks have been well documented to undergo changes upon interaction with saccharides such as glucose.^43,44^ This interaction is the same one responsible for the proposed binding between the 4-MPBA on the SERS platform and the glycan on the cell surface. The peak at 1074 cm^–1^ representing the B–OH stretching undergoes the most significant change upon conversion to a cyclic boronic ester due to the interaction with glycans.^44^ Therefore, the change of signal for this peak was used for the glycan mapping on cell surfaces. The full assignment of 4-MPBA vibrational frequencies is also shown in Table S1† in the ESI.^47^

### Glycan expression of different cell lines probed by SERS

The first SERS based sensor towards glucose sensing was introduced by the Van Duyne group.^48^ A silver film over a nanosphere pattern, where the silver was functionalized with decanethiol was used in this study. More recently, boronic acid-based sensors have been used widely in newer glucose sensing applications using a variety of SERS and fluorescence methods.^49–51^

Boronic acid moieties have also been used for aptasensing of glycoproteins,^52^ and for the detection of glycans on the surface of a cell using fluorescence microscopy.^53^ Glycan expression on the cell surface has further been studied with SERS using a bioorthogonal Raman reporter and lectin-functionalized metallic nanoparticles.^54,55^ These types of nanoparticles have also been shown to be used for the detection of glycans on intact cells and also carbohydrate–protein interactions.^56,57^ However, metallic nanoparticles are hindered by their tendency for heterogeneous distribution and the formation of aggregates, especially on rough surfaces such as cells. As the distribution of glycans on the surface of a cell varies between different cells, it is important to provide a homogenous sensing area beneath or above the cell surface in order to provide more accurate mapping of the expression of glycans on the cell surface. The use of metallic nanoparticles would be beneficial for intracellular studies. However, for studies of cell surfaces, the fabricated platforms described here provide a homogenous sensing surface beneath the cell. This should afford more reproducible and accurate maps of different cellular compartments located near the plasmonic surface and the biomolecules of interest on the surfaces of cells.

Different glycans such as sialoglycans are present on the surfaces of mammalian cells. They play pivotal roles in the regulation of molecular and cellular interactions.^58^ The elevated expression of glycans including the sialic acid containing glycoproteins is indicative of disease and cancer progression.^54,59^ The glycan composition of a cell changes with progression of the cancer. This is attributed to the ability of glycans to prevent cell coagulation and promote rapid entry into the bloodstream to facilitate cancer metastasis.^54,60^ Thus, by tracking the expression of glycans on the plasmonic platform, it can potentially provide a tool to identify cancerous cells.

Upon binding to cell surface glycans, a decrease in the intensities of the two main peaks of 4-MPBA as shown in Fig. 4C (1074 and 1574 cm^–1^) is observed.^44^ The change at 1074 cm^–1^ peak (B–OH stretching) was used to map the locations of glycans on the cell surface. Having demonstrated the successful positioning of cells on the plasmonic platform as described above, three different cell lines were chosen for the evaluation of glycan expression. HEK 293 cells were chosen due to their vast usage in cell biology research for many years and their established cellular growth rate and easy maintenance.^61^ This cell line serves as a control as it expresses no or minimal glycans.^62^ We also used C2C12 mouse myoblast cells. This cell line was selected as a normal mammalian cell line representing a non-diseased state, where normal levels of glycan expression were expected. HeLa cells, the third selected cell line, are derived from cervical tumour cancer cells. This cell line is the oldest and most commonly used human cell line due to its remarkable durability and proliferation and is the first continuous human cancer cell line. Elevated glycan expression is known for cancer cell lines,^63^ and has previously been observed for HeLa cells by SERS.^54^ Although it is possible to do the same experiment on living cells, fixed cells were used in the current study to mitigate any undesired effects due to the movement of cells or changes in glycan expression during the SERS mapping.

**Fig. 5:**
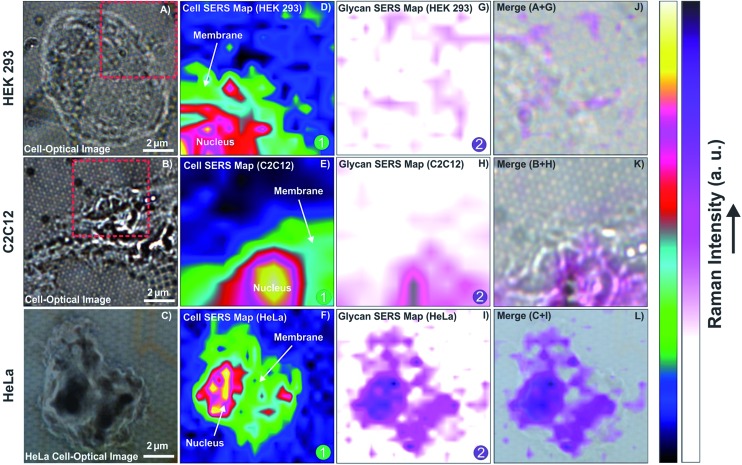
Confocal SERS mapping of cell compartments and glycan expression on HEK 293, C2C12 and HeLa cells. Optical image of isolated single (A) HEK 293 cell, (B) C2C12 cell, (C) HeLa cell; confocal SERS map of cell compartments of selected regions in optical images for (D) HEK 293 cell, (E) C2C12 cell, (F) HeLa cell; confocal SERS map of glycan expression over the selected regions in optical images for (G) HEK 293 cell, (H) C2C12 cell, (I) HeLa cell; (J) overlay of A + G; (K) overlay of B + H; (L) overlay of C + I.

The optical images of randomly selected isolated cells are shown in Fig. 5A–C. In the first step, regions of the isolated cells on the plasmonic platform were mapped by SERS. These maps revealed the major compartments of cells such as the nucleus and membrane as shown in Fig. 5D–F. There are a few overlaps between the Raman reporter and cell vibrational frequencies such as those at ∼1000 cm^–1^ (Fig. 4C). Regardless of these overlaps, by integrating the spectral range of 2800–3000 cm^–1^, one is able to map the major cell compartments of nucleus and cell membrane on the platform as shown in Fig. 5D–F using confocal SERS mapping. By combining AFM and subsequent SERS mapping on a single cell, it is also possible to obtain high resolution images of the cell morphology whilst providing label-free high resolution confocal mapping of cell compartments or a molecule of interest such as glycans on the cell in the presence of the Raman reporter (Fig. S4, ESI†).

The changes in the spectral fingerprint of the Raman reporter (4-MPBA) due to the interaction between 4-MPBA and glycans on the cell surface were used to map the glycan distribution on the cells. After baseline correction and normalization of the data contained within the maps, the average fingerprint of 4-MPBA based on each individual map was selected. This fingerprint was subtracted from each signal obtained on the cell area to map the changes on the cell. It is likely that not every Raman reporter at the surface of the plasmonic platform interacts with glycans, depending on the glycan distribution on the cell. To overcome this problem and to provide a reasonable approach for using the changes in signals associated with Raman reporters interacting with glycans, the average signal of 4-MPBA on the areas that cell is absent is subtracted from the signals obtained on the cell, which are the combination of 4-MPBA and cell signals. The full methodology of the analysis is further explained in detail in the ESI.† By analyzing the data based on the above methodology, the SERS maps of the glycan expression on the cells were generated and are shown in Fig. 5G–I for HEK 293, C2C12 and HeLa cells, respectively.

As shown in Fig. 5G, a minimal change was observed for HEK cells, indicating insignificant expression of glycans on the surface of the cell. The expression of glycans on the C2C12 cell surface is quite clear. In the HeLa cells, the level of glycan expression suggested by the SERS map is significantly higher than that for the C2C12 or HEK 293 cells. This is in agreement with the results of confocal SERS mapping using lectin-functionalized nanoparticles where it was shown that HeLa cells express more glycans than Chinese hamster ovarian (CHO) cells, a cell line also known to exhibit low levels of glycan expression.^54^ The overlay of confocal SERS mapping of glycans on these three cell lines with the corresponding optical images of selected cell surface areas are shown in Fig. 5J–L.

**Fig. 6:**
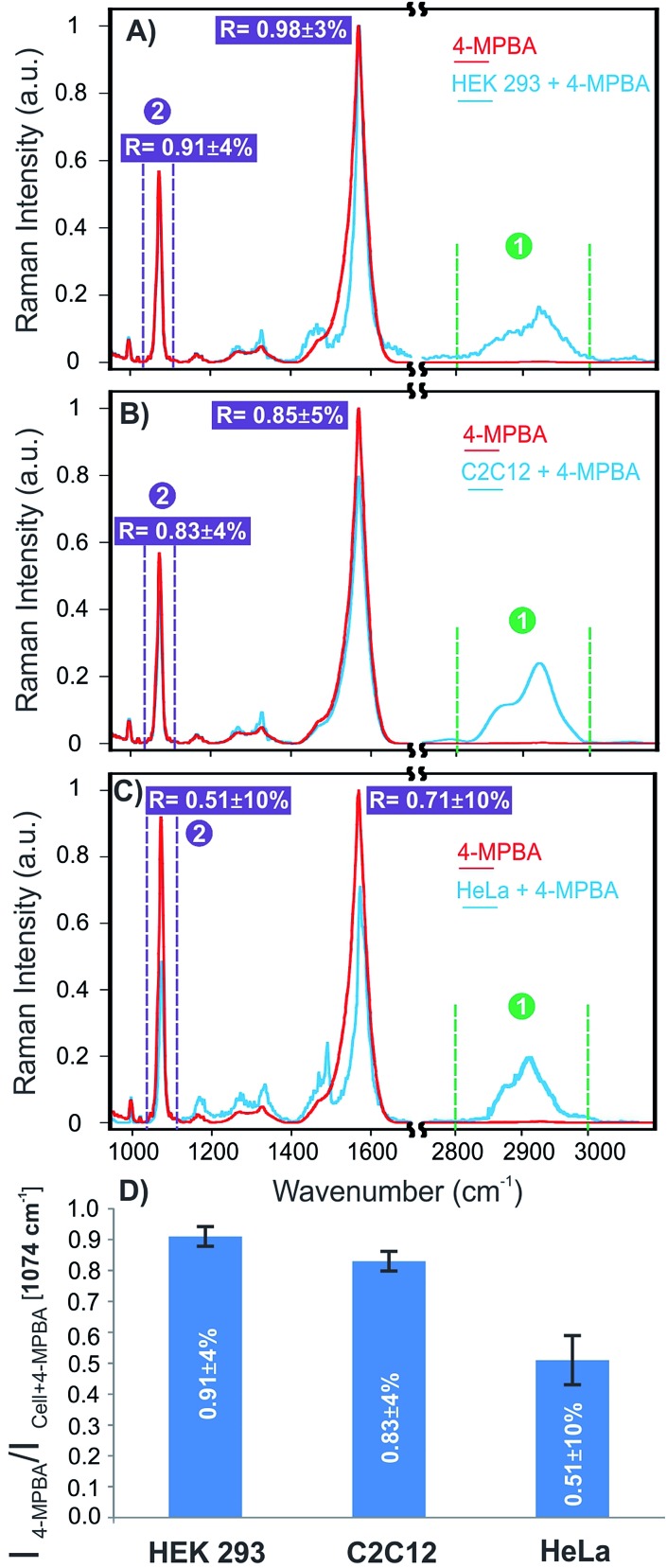
Average SERS spectra of 4MPBA and isolated cells on 4-MPBA functionalized-plasmonic platform; (A) HEK 293 cell; (B) C2C12 cell; (C) HeLa cell; (D) ratios of the average intensities of 4-MPBA/cell + 4MPBA for 15 cells at 1074 cm^–1^. Baseline corrections were applied to all spectra.

The corresponding SERS spectra of 4-MPBA and cells on the platform functionalized with 4-MPBA were also shown for these three cell lines in Fig. 6. To quantify the changes observed for these three cell lines in terms of glycan expression, the ratio of the intensities of the SERS signals of 4-MPBA on the bare plasmonic platform and cells (SERS_intensity_(4-MPBA)/SERS_intensity_(4-MPBA + cell)) were compared at 1074 and 1587 cm^–1^. The full statistical and error analysis of these signals for 15 cells for each cell line are provided in Fig. S5, ESI† showing good consistency and reproducibility of the results for each cell line. As shown in Fig. 6A, the ratios of 0.91 ± 4% and 0.98 ± 3% were observed for the aforementioned signals, respectively. This suggests insignificant change in the Raman signals of 4-MPBA for the HEK 293 cells as a result of no or minimal glycan expression. The ratios obtained for the C2C12 cells were slightly decreased to 0.83 ± 4% and 0.85 ± 5% as shown in Fig. 6B, corresponding to notable expression of glycans, as shown on the SERS map (Fig. 5H). However, a significant change has been observed for HeLa cells representing the ratios of 0.51 ± 10% and 0.71 ± 10% as shown in Fig. 6C. This suggests a distinct elevated expression of glycans on the cell surface compared to C2C12 and HEK 293 cells. The average ratios of 15 cells at 1074 cm^–1^ for these cell lines are shown in Fig. 6D.

Similar observations in terms of the signal changes were reported previously for the 4-MPBA while interacting with glucose.^44^ It has been previously reported that the discrepancies between prostate cancer and non-cancerous cells in terms of glycan expression were much more clearly resolved using SERS compared to fluorescence microscopy. Comparative fluorescence studies only demonstrated a slight difference between the two cell subsets.^54^ This introduces SERS as a critical non-invasive tool to differentiate cancer cells with high sensitivity.

## Conclusions

In conclusion, we have demonstrated the fabrication of a device that provides a promising plasmonic sensing platform for positionally-controlled surface enhanced spectroscopy applications. This platform not only allows one to locate the analyte/reporter in defined positions, but also provides the opportunity to isolate a single cell for analysis of specific biomolecules on their surfaces. The SERS detection of glycan expression in different cell lines including HEK 293, C2C12, and HeLa cells was demonstrated. It was observed that the HeLa cell line derived from cervical cancer cells, expressed more glycans on its surface compared to noncancerous HEK 293 and C2C12 cells. Noteworthy, glycan biosensors have emerged as an alternative to glycan microarrays specifically when sensitivity of analysis is of great importance.^59^ As mentioned, this platform including the integration of plasmonic sensors into micropatterns offers controlled density of cells on the sensing areas for more relevant and accurate statistical studies for acquisition of large data sets and also potential automated measurements. Furthermore, for the glycan studies, cell–cell communication may also affect the glycosylation on the cells specifically in communication of different cultures of cells.^64^ This effect can also be overcome using this platform providing controlled positioning of individual cells on the sensing areas. This strategy may also be further applied to detect proteins on cell surfaces. The SERS platform with ultrasensitive detection capability will be pertinent to study membrane proteins. Raman and infrared vibrations are sensitive to the local environment,^65^ potentially allowing one to probe the conformational changes of cell surface receptors.^55^ This provides the potential applications of the proposed platform to identify other types of cancer cells using SERS. Last but not least, the fabrication process of this platform is entirely compatible with other nanofabrication processes such as electron beam lithography. This only requires alignment control between two consecutive steps, which can be done easily with mask alignment technology. The inclusion of such modified platforms inside microfluidic channel is also possible, highlighting the versatility of the proposed method.^66^
