# Supplementary material for: Controlled positioning of analytes and cells on a plasmonic platform for glycan sensing using surface enhanced Raman spectroscopy
Source: Chem Sci. 2015 Oct 16;7(1):575–82. doi: 10.1039/c5sc03332b (PMC5519955; doi:10.1039/c5sc03332b)
Supplement: Supplementary file 1 [file SC-007-C5SC03332B-s001.pdf]

Electronic Supplementary Material (ESI) for Chemical Science.
This journal is © The Royal Society of Chemistry 2015

Supporting Information

Controlled Positioning of Analytes and Cells on a Plasmonic Platform for Glycan Sensing Application using Surface Enhanced Raman Spectroscopy

Mohammadali Tabatabaei,[a] Gregory Q. Wallace,[a] Fabiana A. Caetano,[b] Elizabeth R. Gillies,[a, c] Stephen S. G. Ferguson,[b] and François Lagugné-Labarthet[a,*]

[a] *Department of Chemistry and Center for Advanced Materials and Biomaterials, University of Western Ontario, London, ON, Canada N6A 5B7*

[b] *J. Allyn Taylor Centre for Cell Biology, Robarts Research Institute, Department of Physiology and Pharmacology, University of Western Ontario, 100 Perth Drive St., London, ON, Canada N6A 5K8*

[c] *Department of Chemical and Biochemical Engineering, The University of Western Ontario, 1151 Richmond Street, London, Ontario, Canada N6A 5B9*

Experimental procedures:

Materials

Standard glass microscope coverslips (22×22×0.15 mm) and microscope slides (25×75×1 mm) were purchased from VWR International and Fisher Scientific, respectively. Plastic dishes (35 mm) with glass bottoms (thickness of 0.16-0.19 mm) were purchased from MatTek Corporation. Nanostrip™ (90% sulfuric acid, 5% peroxymonosulfuric acid, <1% hydrogen peroxide, and 5% water) was purchased from Cyantek Corporation. The materials used in the photolithography process, Microposit™ SC1805™ positive photoresist and Microposit™ MF-319 developer were purchased from Rohm & Haas Electronic Materials, and the NANO™ Remover PG was purchased from MicroChem Corporation. Octafluorocyclobutane gas (C_4F_8) was purchased from BOC Edwards. The wafer mounting medium Crystalbond™ 509 was purchased from Aremco Products, Inc., and finally, the isopropyl alcohol (IPA) and acetone were purchased from Sigma Aldrich and used as received. 1 µm polystyrene microspheres (10% w/w) were purchased from ThermoScientific Co (California, US). Sodium dodecyl sulfate, 4-nitrothiophenol (4-NTP) (80% purity) and 4-mercaptophenylboronic acid (90% purity) were obtained from Sigma-Aldrich (Missouri, US).

Functionalization of the platform

A stock solution of 10^{-3} M 4-nitrothiophenol (4-NTP) in ethanol was prepared. This solution was then diluted to 10^{-6} M. From this solution, two 40 µL aliquots were drop casted onto the surface of a substrate that had been placed into a Petri dish. A clean coverslip was then gently placed onto the top of the substrate to sandwich the uniformly dispersed solution between the substrate and a coverslip. The Petri dish was covered with parafilm and left in the refrigerator to functionalize the surface for 24 hours. After 24 hours, the coverslip was removed, and the substrate was dipped 5 times in 3 beakers of ethanol and then dried under nitrogen. 4-Mercaptophenylboronic acid (4-MPBA) was also dissolved in ethanol to form a 10 mM solution for functionalization of the platform as the Raman reporter for glycan study with the same procedure as 4-NTP.

SERS setup

The SERS measurements were performed using a Horiba Jobin-Yvon Labram HR Raman microspectrometer equipped with a 600 grooves/mm grating and a 632.8 nm excitation. The intensity was set to 1 mW at the sample. A microscope objective of 100X, 0.9 N.A. was used for all experiments. The pinhole of the spectrometer was opened to 200 µm. An acquisition time of 30 s with 5 accumulations was used for spectra shown in Figure 5. SERS maps ranged in size from as small as 5 µm × 5 µm to as large as 30 µm × 30 µm. A step-size of 1 µm was used for the SERS maps as this corresponds to the approximate size of the laser spot. For the construction of the SERS map shown in Figure 3, the acquisition time of each individual spectrum was set to 1 s. An acquisition time of 10 second/spectrum is used for all the other SERS maps. SERS mapping was conducted on 15 cells for each cell line for statistical purposes.

Atomic force microscopy (AFM)

AFM images were collected with a Bioscope Catalyst instrument (Bruker), operating in contact mode. A silicon cantilever with a spring constant of 40 N m^{-1} and an oscillating frequency of 325 kHz (NSC-15, Micromasch) was used.

Scanning electron microscopy (SEM)

SEM images were obtained using a LEO Zeiss 1530 (Zeiss, Oberkochen, Germany). Prior to imaging, substrates were coated with a 5 nm thick layer of osmium.

Cultures of HEK 293, C2C12, and HeLa cells

HEK 293, C2C12, and HeLa cells were maintained in glucose-free Eagle's Minimal Essential Medium (MEM, Invitrogen). They were then supplemented with 2 mM glutamine (used just for C2C12), 50 µg mL^{-1} gentamicin (Invitrogen), and 10% Fetal Bovine Serum (Invitrogen). The cultures were kept in an incubator at 37 °C, 5% CO_2, 100% humidity, and were passaged every 3

days by trypsinization (0.25% trypsin-EDTA, Invitrogen). The cells were seeded onto the patterned substrates at a density of 8×10^5 cells/dish and incubated for 48 h before fixation.

Culture of neuronal cells

Cortical neuron cells (CD-1 strain mice, 14-15 day embryonic age) were also used. Primary cortical neurons were dissociated in Hank's balanced salt solution (HBSS) and plated in Neurobasal™ media supplemented with 0.5 mM L-glutamine, 2% B_{27} and 0.8% N_2 supplements, and 50 units mL^{-1} penicillin-streptomycin (Invitrogen). Cultures were then plated onto the patterned surfaces at a density of 1×10^6 cells/dish. The cultures were incubated at 37 °C for 14 days. The media was also changed every 2-3 days. Animal handling protocol was in accordance with Western University (The University of Western Ontario) Animal Care Committee. All of the cell lines and cortical neurons were fixed using a solution of 4% paraformaldehyde and sucrose prepared in phosphate buffered saline (PBS, pH 7.4, Invitrogen).

Fabrication process of FC-patterned plasmonic platform

Microscope coverslips were cleaned in Nanostrip™ for 5 min and rinsed thoroughly with de-ionized (DI) water prior to use. The fabrication process, as outlined in Fig. 1, was performed in four main steps. This includes NSL using an interface method, photolithography, plasma polymerization, followed by a lift-off process. NSL using the interface method was previously described in detail.[1-3]

Patterning of the plasmonic platform using optical lithography employed a patterned photoresist mask to protect regions of the plasmonic substrate. A positive photoresist was spin-coated onto the cleaned plasmonic substrate, with a thickness of approximately 500 nm, as measured by atomic force microscopy.[4] For the smaller features in range of 5 µm or less, use of a thinner layer of photoresist led to sharper patterns. The photoresist was then soft-baked at 115 °C for 1 min to evaporate the solvent, followed by baking in an oven at 90 °C for 20 min. It was then exposed to ultra-violet light through a chrome mask that had the desired pattern designed on it. For this purpose, a Karl Suss MA6 contact mask aligner (Suss MicroTech) was used for 8 s at an intensity of 12 mW cm^{-2}. The exposed photoresist was then removed by developing in Microposit MF-319 developer for 1 min. It was then washed with DI water and dried under nitrogen. The photoresist mask left on the surface is a replication of the original optical mask. Subsequently, the plasma deposition of the fluorocarbon polymer was carried out using a deep reaction ion etching instrument, and inductively coupled plasma (ICP) reactor, (Alcatel 601E). The source power and substrate bias were set to 1,800 and 80 W, respectively. The feed gas supplied was C_4F_8, controlled at a flow of 150 sccm [standard cubic centimetres per minute] with a total process time of 20 s at a temperature of 20 °C. The photoresist-

patterned plasmonic platform fabricated on coverslips was mounted onto silicon wafers for the plasma deposition and were subsequently removed prior to the lift-off process. A uniform layer of approximately 60 ± 5 nm (Fig. S1, ESI) of fluorocarbon polymer was deposited on the plasmonic platform. Finally, the protective photoresist was removed by a lift-off process in a photoresist solvent (NANO^{TM} Remover PG) for 10 min at 80 °C, followed by sonication in the solvent for 30 s. The sample was then rinsed thoroughly with isopropyl alcohol and dried under nitrogen. The final architecture of the substrate is a thin fluoropolymer film (60 ± 5 nm) with regions wherein the nanotriangular plasmonic platform is revealed.

AFM characterization of the FC-patterned substrate

To verify the thickness of deposited FC polymer film on the substrates, AFM topography images were collected. These samples were used as control samples next to the NSL samples at the same time of mounting for plasma-induced FC polymer deposition.

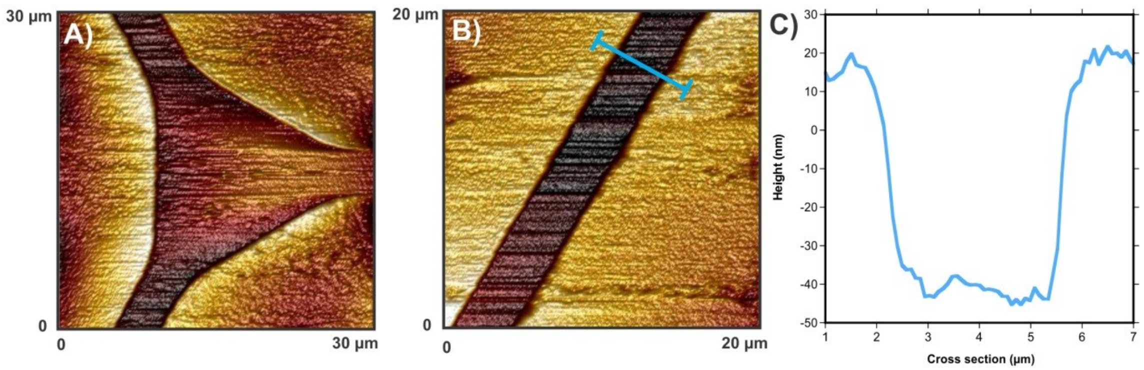

Fig. S1. AFM topography of A) a node and B) a channel of a hexagonal grid-like FC-patterned substrate on glass coverslip. C) Cross section of the indicated area in B determining the thickness of FC film of 60 ± 5 nm.

Controlled micro-defined functionalization with analyte

In order to evaluate the confinement of the analyte by the patterned fluoropolymer thin film, as well as the SERS activity of the embedded plasmonic platform, the sensing device was functionalized with 4-nitrothiophenol (4-NTP).

A schematic representation of the functionalized platform is shown in Fig. S2A. 4-NTP binds specifically to gold via its thiol moiety, but not to the FC film. After a 24 h functionalization in a 1 µM solution of 4-NTP in ethanol, the substrates were gently rinsed with ethanol. SERS mapping was performed over the sensing area to evaluate the surface enhanced activity on the gold nanotriangles and the FC polymer-coated regions. A SERS map of 4-NTP on a single triangular pattern is shown in Fig. S2B. This map

corresponds to the NO_2 peak (1337 cm^{-1}) of 4-NTP and was obtained from the integration over the region from 1300-1350 cm^{-1}. Selected steps of 1 μm and an acquisition time of 1 s/spectrum under 632.8 nm excitation were sufficient to probe the monolayer of 4-NTP. The SERS spectrum from a pixel found within the nanotriangles inside the pattern (labelled as 1 in Fig. S2B) shows a well-defined SERS spectrum of 4-NTP (Fig. S2C).[5, 6] On the other hand, the SERS spectrum of the fluoropolymer region (labelled as 2 in Fig. S2B) shows only broad, low intensity background peaks likely corresponding to the fluoropolymer itself, due to its low scattering cross section. This suggests that binding of 4-NTP and consequent SERS enhancement of the reporter are confined to the defined regions where the gold plasmonic platform is exposed. The optical image of a hexagonal grid-like pattern on the plasmonic substrate is shown in Fig. 3D. The selected regions for mapping are shown in red. SERS mapping areas were selected around the features of interest as 22×30, 30×30, and 10×10 μm^2 for Fig. S2 B, E, and F, respectively. Fig. S2 E and F show SERS maps for 4-NTP that were obtained using the same procedure described above.

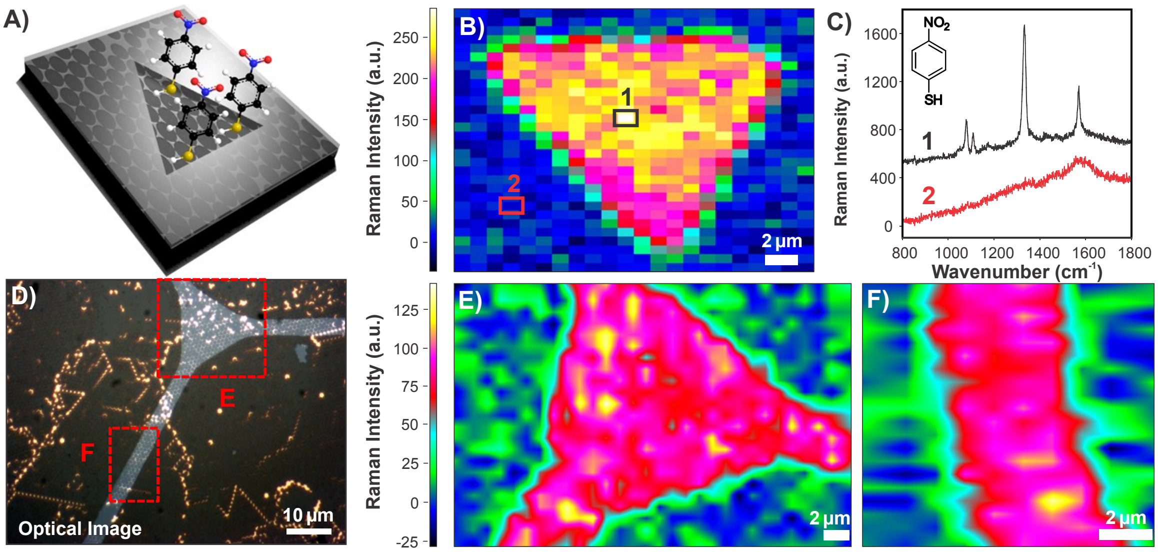

Fig. S2. Collected confocal SERS spectra and mapping of functionalized 4-NTP on a FC-patterned plasmonic substrate. A) Schematic of the microwell plasmonic platform B) SERS mapping on an isolated triangular pattern C) Chemical structure of 4-NTP and collected SERS spectra of defined regions in B. Region 1 and 2 are located on plasmonic platform and FC polymer, respectively. D) Optical image of a node and channels positioned over the NSL substrate. SERS mappings of selected regions in D representing a node (E) and a channel (F) are shown in the interpolated images of E and F.

These results demonstrate the ability of the patterned plasmonic platforms to provide positionally-controlled functionalization with specific molecules for sensitive detection.

This is consistent with previous results demonstrating the ability of plasmonic platforms to provide ultrasensitive detection down to sub-femtomolar concentration.[3, 7-9]

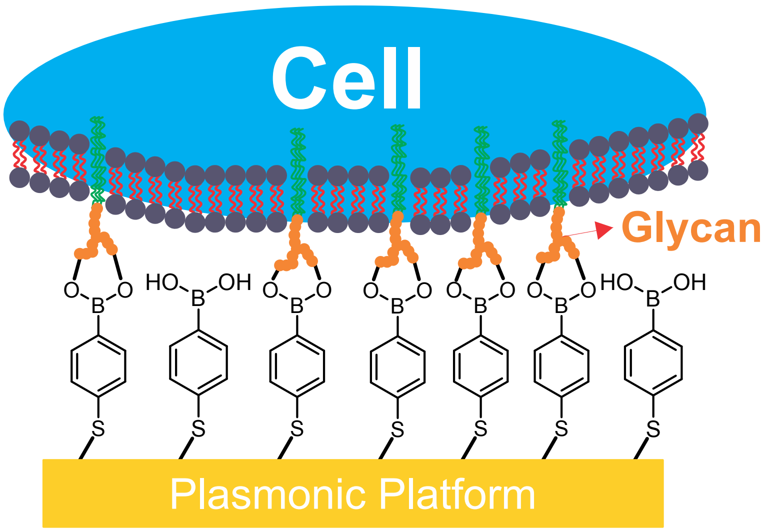

Fig. S3. Schematic representation of interaction between glycans and the Raman reporter (4-MPBA) on plasmonic substrate.

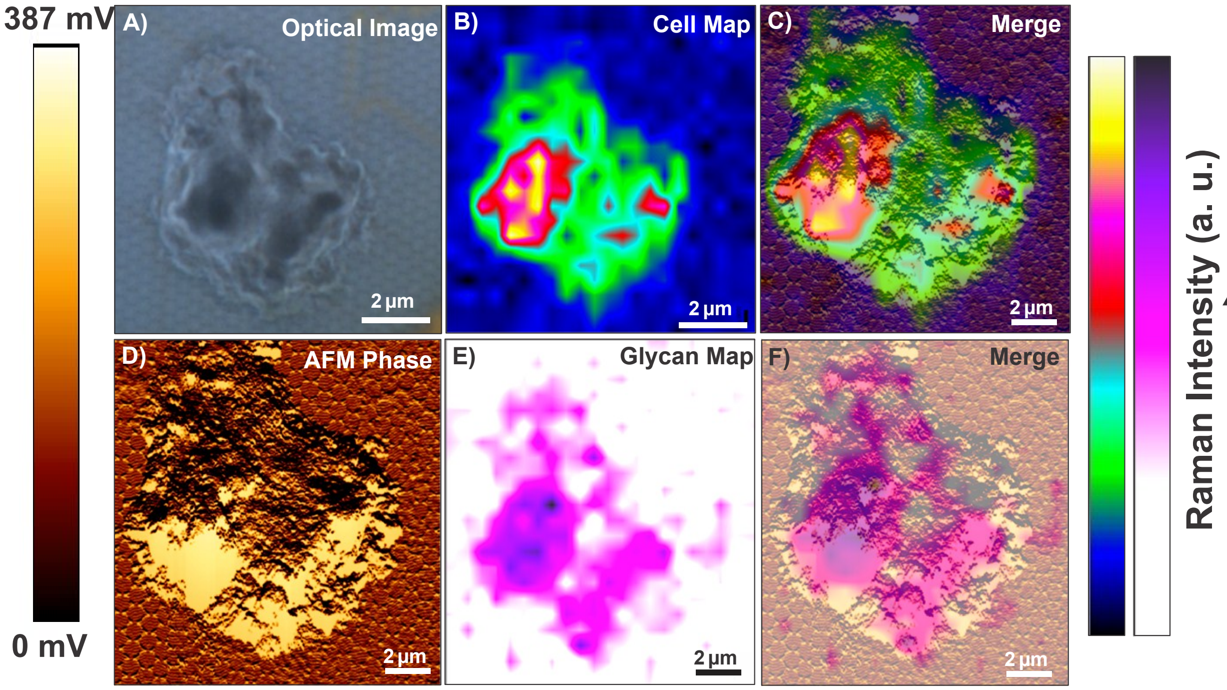

Fig. S4. Confocal SERS mapping of a single HeLa cell with nanoscale surface morphology obtained by AFM. A) Optical image of a single HeLa cell; B) SERS mapping of cell compartments;

C) Overlay of SERS map in B with AFM morphology shown in D; D) AFM phase of the single cell shown in D. E) Glycan distribution on the same cell; F) Overlay of D and E.

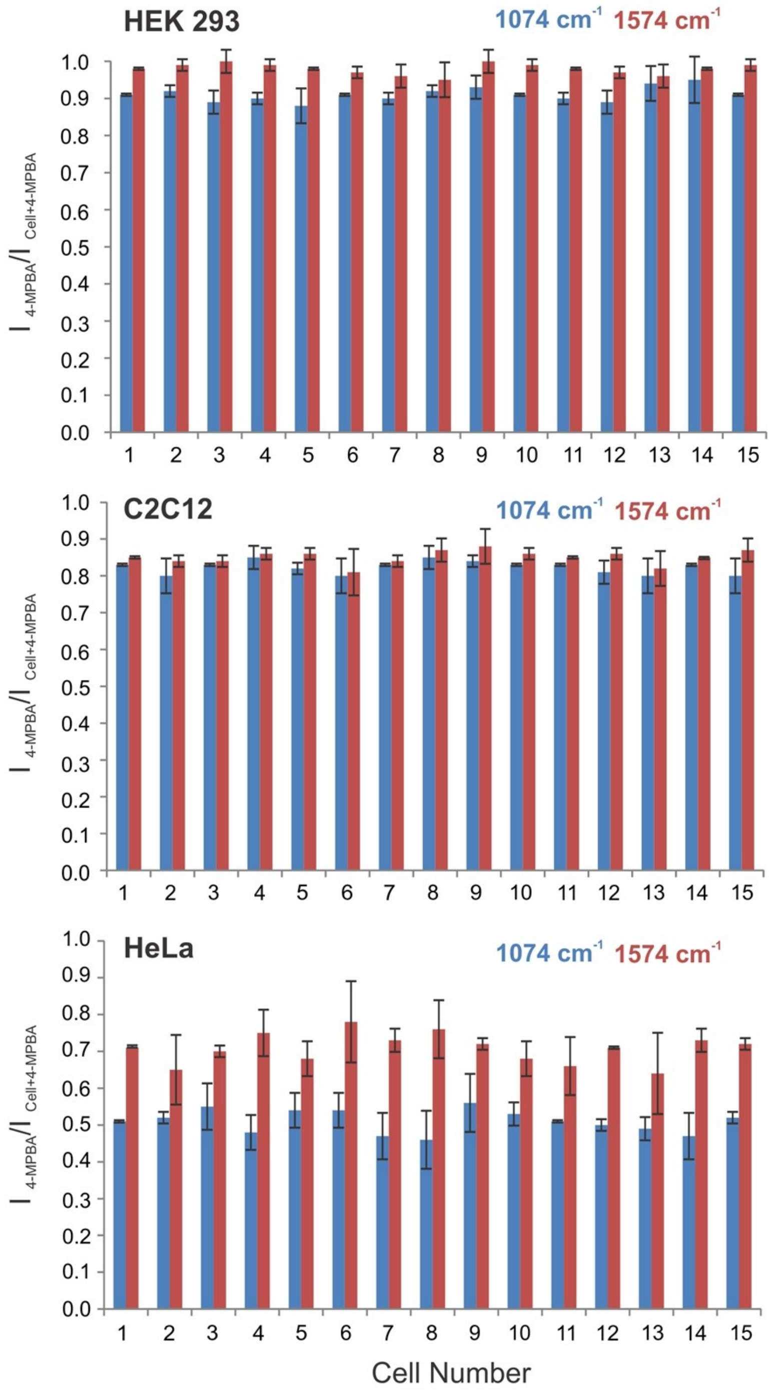

Fig. S5. Cell SERS analysis representing the ratios of the normalized average SERS (Intensity (4-MPBA)/Intensity (4-MPBA + Cell)) at 1074 and 1574 cm^{-1} for 15 different cells for each cell line. The error bars are generated based on the percent deviation from the median ratio values. The

obtained median values are 0.91 ± 4% (HEK 293 at 1074 cm^{-1}), 0.98 ± 3% (HEK 293 at 1574 cm^{-1}), 0.83 ± 4% (C2C12 at 1074 cm^{-1}), 0.85 ± 5% (C2C12 at 1574 cm^{-1}), 0.51 ± 10% (HeLa at 1074 cm^{-1}), 0.71+10% (HeLa at 1574 cm^{-1}).

Table S1. SERS vibrational frequencies assignment of 4-MPBA and Cell[10-16]

4-MPBA SERS Peaks (cm^{-1})	Assignment	Cell SERS Peaks (cm^{-1})	Assignment
1000	δ_{C-C-C}	1003	phenylalanine of protein and lipid
1020	ν_{C-S}	1091	symmetric dioxy stretch of the phosphate backbone and ν_{C-C} of nucleic acids lipid
1074	ν_{B-OH}	1448	δ_{CH2} proteins and lipids
1182	ν_{B-C}	1651	amide $\nu_{C=O}$ and $\nu_{C=C}$ of protein and unsaturated lipid
1472	$\nu_{C=C}$	2845<br>2875	ν_{CH2} of protein and lipid
1574	ν_{C-C}	2932	
δ (Bending); ν (Stretching)			

SERS signal analysis for glycan mapping

After collecting the SERS maps, there are few analysis steps that must be applied to the mapping before generating the final glycan mapping. The first step was to apply the baseline correction and normalization on all of obtained signals. Next, it was necessary to differentiate the signals coming from the 4-MPBA alone and 4-MPBA+cell. By looking at the spectral range between 2800 and 3000 cm^{-1}, we can distinguish the peaks that contain the cell components. After normalization, each pixel of the map that contained the cell peak in this range was excluded. This provided all of the pixels that contained the signal of 4-MPBA alone. The average signal of these pixels gave the average signal of 4-MPBA without the presence of cell. When 4-MPBA as the Raman reporter was attached to the glycan on the cell surface, a change occurred in its signals significantly decreasing the intensities of the peaks at 1074 and 1574 cm^{-1}. By

subtracting the average 4-MPBA signal obtained previously from all of the pixels on the map, it was possible to obtain the changes to signal collected from the Raman reporter attached to the glycans over the cell surfaces. By integrating the area under the peak of 1074 cm^{-1}, the glycan mapping over cells as shown in Fig. 5 G-I was generated. The average signal of 4-MPBA alone and also the average signal of collected signals on the cell area including the 4-MPBA and cell are illustrated in Fig.6 to show the expected differences obtained while the Raman reporter was attached to the glycans on the cell surfaces. The statistical and error analysis for 15 randomly selected cells of each cell line were also performed for the changes to the signals at 1074 and 1574 cm^{-1} and are shown in Fig. S5. All of the maps were normalized to the same scale for the sake of comparison between different cell lines.

References:

1. S. Fayyaz, M. Tabatabaei, R. Hou and F. Lagugné-Labarthet, *J. Phys. Chem. C*, 2012, **116**, 11665-11670.
2. M. Tabatabaei, A. Sangar, N. Kazemi-Zanjani, P. Torchio, A. Merlen and F. Lagugné-Labarthet, *J. Phys. Chem. C*, 2013, **117**, 14778-14786.
3. G. Q. Wallace, M. Tabatabaei and F. Lagugné-Labarthet, *Can. J. Chem.*, 2014, **92**, 1-8.
4. M. Tabatabaei, F. A. Caetano, S. Vedraine, P. R. Norton, S. S. G. Ferguson and F. Lagugné-Labarthet, *Biomaterials*, 2013, **34**, 10065-10074.
5. V. Kiran and S. Sampath, *ACS Appl. Mater. Interfaces*, 2012, **4**, 3818-3828.
6. B. Dong, Y. Fang, L. Xia, H. Xu and M. Sun, *J. Raman Spectrosc.*, 2011, **42**, 1205-1206.
7. F. De Angelis, F. Gentile, F. Mecarini, G. Das, M. Moretti, P. Candeloro, M. L. Coluccio, G. Cojoc, A. Accardo, C. Liberale, R. P. Zaccaria, G. Perozziello, L. Tirinato, A. Toma, G. Cuda, R. Cingolani and E. Di Fabrizio, *Nat. Photon.*, 2011, **5**, 682-687.
8. M.-W. Shao, L. Lu, H. Wang, S. Wang, M.-L. Zhang, D.-D.-D. Ma and S.-T. Lee, *Chem. Commun.*, 2008, DOI: 10.1039/B802405G, 2310-2312.
9. M. Tabatabaei, M. Najiminaini, K. Davieau, B. Kaminska, M. R. Singh, J. J. L. Carson and F. Lagugné-Labarthet, *ACS Photonics*, 2015, **2**, 752–759.
10. X. Zhang, M. B. J. Roeffaers, S. Basu, J. R. Daniele, D. Fu, C. W. Freudiger, G. R. Holtom and X. S. Xie, *Chem. Phys. Chem.*, 2012, **13**, 1054-1059.
11. H. Torul, H. Ciftci, F. C. Dudak, Y. Adguzel, H. Kulah, I. H. Boyac and U. Tamer, *Anal. Methods*, 2014, **6**, 5097-5104.
12. M. Rycenga, J. M. McLellan and Y. Xia, *Chem. Phys. Lett.*, 2008, **463**, 166-171.
13. N. Kanayama and H. Kitano, *Langmuir*, 2000, **16**, 577-583.
14. Y. Erdogdu, M. Tahir Guellueolu and M. Kurt, *J. Raman Spectrosc.*, 2009, **40**, 1615-1623.
15. M. Kurt, T. Raci Sertbakan, M. Ozduran and M. Karabacak, *J. Mol. Struct.*, 2009, **921**, 178-187.
16. F. Sun, T. Bai, L. Zhang, J.-R. Ella-Menye, S. Liu, A. K. Nowinski, S. Jiang and Q. Yu, *Anal. Chem.*, 2014, **86**, 2387-2394.
